# A Pilot Study on MicroRNA Profile in Tear Fluid to Predict Response to Anti-VEGF Treatments for Diabetic Macular Edema

**DOI:** 10.3390/jcm9092920

**Published:** 2020-09-10

**Authors:** Hwei Wuen Chan, Binxia Yang, Wendy Wong, Paul Blakeley, Ivan Seah, Queenie Shu Woon Tan, Haofei Wang, Mayuri Bhargava, Hazel Anne Lin, Charmaine HC Chai, Erlangga Ariadarma Mangunkusumo, Naing Thet, Yew Sen Yuen, Raman Sethi, Si Wang, Walter Hunziker, Gopal Lingam, Xinyi Su

**Affiliations:** 1 Department of Ophthalmology, National University Hospital, Singapore S118177, Singapore; hwei_wuen_chan@nuhs.edu.sg (H.W.C.); wendy_wong@nuhs.edu.sg (W.W.); ivan.seah@mohh.com.sg (I.S.); mayuri_bhargava@nuhs.edu.sg (M.B.); hazel_anne_lin@nuhs.edu.sg (H.A.L.); charmaine_hc_chai@nuhs.edu.sg (C.H.C.); erlangga_ariadarma@nuhs.edu.sg (E.A.M.); naing_thet@nuhs.edu.sg (N.T.); yew_sen_yuen@nuhs.edu.sg (Y.S.Y.); gopal_lingam@nuhs.edu.sg (G.L.); 2 Department of Ophthalmology, Yong Loo Lin School of Medicine, National University of Singapore, Singapore 119228, Singapore; Paul_Blakeley@imcb.a-star.edu.sg (P.B.); ophwsi@nus.edu.sg (S.W.); 3 Institute of Molecular and Cell Biology (IMCB), Agency for Science, Technology and Research (A*STAR), Singapore 138673, Singapore; yangbx@imcb.a-star.edu.sg (B.Y.); swtan@imcb.a-star.edu.sg (Q.S.W.T.); hfwang@email.unc.edu (H.W.); ophrs@nus.edu.sg (R.S.); hunziker@imcb.a-star.edu.sg (W.H.); 4 Singapore Eye Research Institute (SERI), Singapore National Eye Centre, Singapore 169856, Singapore

**Keywords:** anti-vascular endothelial growth factor, aflibercept, bevacizumab, biomarker, diabetic macular edema, microRNA

## Abstract

(1) Background: Intravitreal anti-vascular endothelial growth factor (anti-VEGF) is an established treatment for center-involving diabetic macular edema (ci-DME). However, the clinical response is heterogeneous. This study investigated miRNAs as a biomarker to predict treatment response to anti-VEGF in DME. (2) Methods: Tear fluid, aqueous, and blood were collected from patients with treatment-naïve DME for miRNA expression profiling with quantitative polymerase chain reaction. Differentially expressed miRNAs between good and poor responders were identified from tear fluid. Bioinformatics analysis with the miEAA tool, miRTarBase Annotations, Gene Ontology categories, KEGG, and miRWalk pathways identified interactions between enriched miRNAs and biological pathways. (3) Results: Of 24 participants, 28 eyes received bevacizumab (15 eyes) or aflibercept (13 eyes). Tear fluid had the most detectable miRNA species (*N* = 315), followed by serum (*N* = 309), then aqueous humor (*N* = 134). MiRNAs that correlated with change in macular thickness were miR-214-3p, miR-320d, and hsa-miR-874-3p in good responders; and miR-98-5p, miR-196b-5p, and miR-454-3p in poor responders. VEGF-related pathways and the angiogenin-PRI complex were enriched in good responders, while transforming growth factor-β and insulin-like growth factor pathways were enriched in poor responders. (4) Conclusions: We reported a panel of novel miRNAs that provide insight into biological pathways in DME. Validation in larger independent cohorts is needed to determine the predictive performance of these miRNA candidate biomarkers.

## 1. Introduction

Diabetic macular edema (DME) is the most common sight-threatening complication of diabetes afflicting working-age adults [1,2]. This condition is characterized by fluid accumulation at the macula, which results in the loss of clear central vision needed for reading, driving, and recognizing faces [2]. The estimated 10-year incidence of developing DME is 20% for individuals with type 1 diabetes mellitus (DM) and 25% for those with type 2 DM [2,3,4]. The pathophysiology of DME is complex, mediated by both vascular endothelial growth factor (VEGF) and proinflammatory cytokines. The resultant breakdown of the blood–retinal barrier culminates in vascular leakage and fluid accumulation at the macula.

Since the advent of anti-VEGF therapy, various landmark studies have demonstrated the efficacy of intravitreal anti-VEGF agents in improving the best corrected visual acuity (BCVA) and retinal central subfield thickness (CST) of patients with DME [5,6]. Currently, the mainstay anti-VEGF agents for DME treatment include Bevacizumab (Avastin^®^), Ranibizumab (Lucentis^®^), and Aflibercept (Eylea^®^). However, the response of DME patients to the various anti-VEGF agents varies, and approximately 40% of DME patients may have a suboptimal response to anti-VEGF treatment [7]. It would be desirable if clinicians were able to identify a patient’s propensity to respond to anti-VEGF either through a retinal imaging or biochemical biomarker. Although attempts have been made to predict a patient’s response to anti-VEGF treatments using demographic and/or imaging characteristics, these tools lack validation and remain to be adopted into clinical practice [8,9]. Therefore, poor response is usually ascertained through a prolonged trial of treatment with multiple injections [8,10,11]. This approach is undesirable, as ineffective treatment subjects patients to disease progression, additional costs related to injections, and an incremental risk of sight-threatening complications such as endophthalmitis. Furthermore, patients who do not respond to anti-VEGF agents may benefit from early commencement of slow-release corticosteroid injection such as Dexamethasone implant (Ozurdex^®^). Ideally, this switch should be done as soon as possible to preserve the best visual acuity outcome. Hence, this highlights the need for a noninvasive method to predict likely therapeutic response to currently available anti-VEGF treatments.

MicroRNAs (miRNA) are small noncoding RNAs approximately 18–25 nucleotides in length. miRNAs were initially thought to exist intracellularly. In the cell, they reduce target gene expression via either post-transcriptional regulation of messenger RNA (mRNA) degradation or inhibition of translation [12,13,14]. However, they were eventually detected extracellularly in serum, plasma, urine and saliva, tears, aqueous humor, and vitreous humor [15,16,17,18]. This is because the stability of miRNAs can be preserved in exosomes, microvesicles, or carrier protein complexes [19,20]. Circulating exosomal miRNAs have been shown to mediate paracrine and endocrine tissue signaling with the capacity to modulate gene expression and function of distal cells [21]. Changes in the levels of extracellular miRNAs have been associated with ongoing pathological processes in the body, such as oncogenesis, angiogenesis, and metabolic dysfunction such as diabetes [21,22]. As such, there has been increased interest in harnessing these miRNAs as biomarkers for the diagnosis and prognostication of various diseases. The suitability of miRNAs as biomarkers is further supported by the fact that miRNAs can remain stable after undergoing several freeze-thaw cycles, large variations in pH, and prolonged exposure to room temperature [15,19,23].

From the ocular perspective, miRNAs found in ocular tissue have been shown to display tissue-specific expression patterns, with essential roles in ocular development and retinal homeostasis [24,25,26,27,28]. Studies of the retina from diabetic murine animal models have also suggested the role of several miRNAs in the pathogenesis of DME, including VEGF expression regulation and blood-retinal barrier homeostasis [29,30,31]. Although miRNAs can be detected from the ocular fluids, such as the aqueous humor, vitreous humor, and tear fluid [15,20,21], the utility of miRNAs in tear fluid as potential biomarkers has not been fully explored. In addition, there has been no study so far on the comparison of the miRNA profiles between tear fluid with other ocular fluids like aqueous or vitreous humor. Tears fluids are of particular interest as they can be collected noninvasively without sophisticated equipment. This allows clinicians to conduct a comfortable, low-risk, point-of-care test.

In this study, we performed a systematic comparison of the miRNA profiles generated from tears, aqueous, and serum and identified potential miRNAs in tears to differentiate between responders and suboptimal responders to anti-VEGF therapy.

## 2. Materials and Methods

### 2.1. Study Design

This prospective, single-center interventional case series was conducted with National Healthcare Group Domain Specific Institutional Review Board approval (reference 2015/00344), and adhered to the tenets of the Declaration of Helsinki. Written informed consent was provided by all study participants.

Study participants were at least 21 years of age, had type 1 or 2 diabetes and treatment naïve DME causing visual loss, with Snellen visual acuity of 20/32 or worse, and retinal thickness greater than 300 μm in the central subfield with retinal thickening on spectral-domain optical coherence tomography (SD-OCT) in the study eye. Patients with pan-retinal laser photocoagulation within three months, major intraocular surgery within four months, or concomitant ocular disease that may directly or indirectly cause macular edema, were excluded.

### 2.2. Treatment of DME and Assessment of Treatment Response

A total of 24 patients were recruited for this study. Study participants received either intravitreal Bevacizumab (1.25 mg/0.05 mL) or Aflibercept (2.0 mg/0.05 mL) at baseline, then every four weekly injections unless there was complete resolution of retinal thickening on SD-OCT. For patients who had both eyes recruited for the study, the same anti-VEGF agent was administered in both eyes.

Treatment response was assessed based on the Diabetic Retinopathy Clinical Research Network (DRCR.net) Protocol T SD-OCT morphological criteria, and the % change in central subfield thickness (CST) between baseline and at 12 weeks [32]. Good response was defined as a 20% or greater reduction in CST. Partial response was defined as between 10% to 20% reduction in CST. Poor response was defined as less than 10% reduction in CST or an increase in CST. In three cases wherein the pre-injection CST was close to 300 microns, the reduction in CST could be less than 20%, but the foveal contour could be normalized. In these cases, we selected a juxta foveal point of maximal thickness for the calculation of the reduction in CST (by decentering the ETDRS (Early Treatment in Diabetic Retinopathy Study) grid). This decision was taken only if all the three graders (CHW, WW, GL) agreed.

### 2.3. Collection of Patient Specimens

Three biofluids—tears, aqueous, and blood—were collected only from the first six patients (Batch 1): 20 mL of blood, 100 µL to 200 µL of aqueous, and two Schirmer’s strips of tears at baseline and at the fourth visit (after receiving three consecutive intravitreal injections). For the remaining 18 patients (Batch 2), only tear samples were collected at the baseline visit prior to the first intravitreal injection and at the fourth visit (after receiving three consecutive intravitreal injections). All samples were snap-frozen at −80 degrees Celsius until analysis was performed.

### 2.4. RNA Extraction and Reverse Transcription

Total RNA was extracted using the QIAGEN miRNeasy extraction kit following the instruction. Extracted RNA with spike-in controls were reverse transcribed and underwent multiplex augmentation by touch-down amplification to increase the amount of cDNA without changing the total miRNA levels.

All spike-in controls were synthetic miRNA mimics (22–24 bases of small single-stranded RNAs) designed in silico for low-sequence similarity to all known human miRNAs, thus minimizing cross-hybridization to the primers used in the assays. The spike-in controls serve to detect the presence of inhibitors and correct for technical variations during miRNA isolation, reverse transcription, augmentation, and qPCR. Synthetic miRNA standards of known concentration were diluted over at least six orders of magnitude, amplified, and generated a standard curve for a copy number determination of samples for each miRNA assay. The standard curve was used to further correct for technical variation and to assess the efficiency of each miRNA assay in every PCR plate. This ensured the reliability of the assay. The miRNA assays were judiciously divided into several multiplex groups in silico to minimize nonspecific amplification and primer–primer interactions.

### 2.5. MicroRNA Expression Quantification Using qPCR Assay

The augmented cDNA was diluted and quantified using an SYBR Green-based single-plex qPCR assay (MiRXESTM, Singapore) on Applied Biosystems^®^ ViiA 7 384 Real-Time PCR System (Life Technologies, Singapore).

The raw Cycles to Threshold (Ct) values were processed and the absolute copy numbers of the target miRNAs in each sample were determined by interpolation of standard curves derived from synthetic miRNA. MiRNAs at ≤500 copies/mL fall close to the detection limit of the single-plex qPCR assay (≤10 copies/well). Such low levels were considered undetectable and excluded from analyses. Raw qPCR data was normalized in two steps: Technical and global normalization. The technical variations introduced during RNA isolation and RT-qPCR were normalized by the spike-in control. This was followed by performing global unsupervised analysis and principal component analysis (PCA) on data from all detected miRNAs with adjustment by global normalization to equalize the distribution of miRNA expression across samples.

### 2.6. Bioinformatics and Statistical Analysis of miRNA Expression

Global mean normalized data were used to compare miRNA detection and expression between the three biofluids. Hierarchical clustering of miRNA expression and sample-wise Pearson correlation were performed in *R* and visualized using heatmaps generated by the package “pheatmap”. Boxplots comparing median expression and number non-detects between the three biofluids were generated using ggplot2, in which upper and lower box hinges correspond to the first and third quartiles, respectively. The *R* package “prcomp” was used to perform principal component analysis using both the scaling and centering options.

Quantile normalization was performed to remove batch to batch variations in RT qPCR data. The mixOmics implementation [33,34] of the Sparse Projection to Latent Structures Discriminant Analysis (sPLS-DA) algorithm was used to identify differential miRNA expression between good, partial, and poor responders. sPLS-DA utilizes variable selection, dimension reduction, and k-fold cross-validation, and was used to identify a subset of miRNAs that are differentially expressed between good and poor responders. A separate analysis using a sPLS-DA model on good, partial versus poor responders was used to generate loading scores for all miRNAs, indicating the degree of confidence of differential expression for each miRNA.

The unpaired two-sample Wilcoxon test in *R* was used to calculated *p*-values for differential miRNA expression between poor and good responders. These were compared with sPLS-DA loading scores using a volcano plot, and the 30 miRNAs selected by sPLS-DA were highlighted. Differentially expressed miRNAs between good and poor responders, identified by sPLS-DA, were investigated for signaling pathways enrichment in good or poor responders using the microRNA Enrichment Analysis and Annotation tool (miEAA) [35]. miEAA was run using the Gene Set Enrichment Analysis option and four databases were searched: miRTarBase Annotations [36], Kyoto Encyclopedia of Genes and Genomes (KEGG) Pathways [37], miRWalk Pathways [38], and Gene Ontology (GO) Pathway [39,40]. Ontology terms with a significant *p*-value (<0.01) and an overlap of at least three miRNAs were retained and generic or redundant terms were excluded. The KEGG Pathway Search Tool [41] was used to construct pathway diagrams showing the interactions between the enriched miRNAs and individual signaling pathway components.

## 3. Results

### 3.1. MiRNA Abundancy Was Significantly Higher in Tears Compared to Aqueous Humor

In this study, the first 6 patients had all the three biofluids (tears, aqueous and blood) collected for miRNA studies, while the subsequent 18 patients had only tears collected. To evaluate the suitability of tears for miRNA profiling, all three biofluid samples from six patients in Batch 1 collected at baseline (prior to treatment) were analyzed and compared. Twelve samples of each type of biofluid (six from baseline and six post-treatment) underwent miRNA profiling using a qPCR-based absolute quantification method. The absolute copy number of miRNAs identified in the different types of biofluids was used to evaluate their suitability for biomarker discovery.

**Figure 1:**
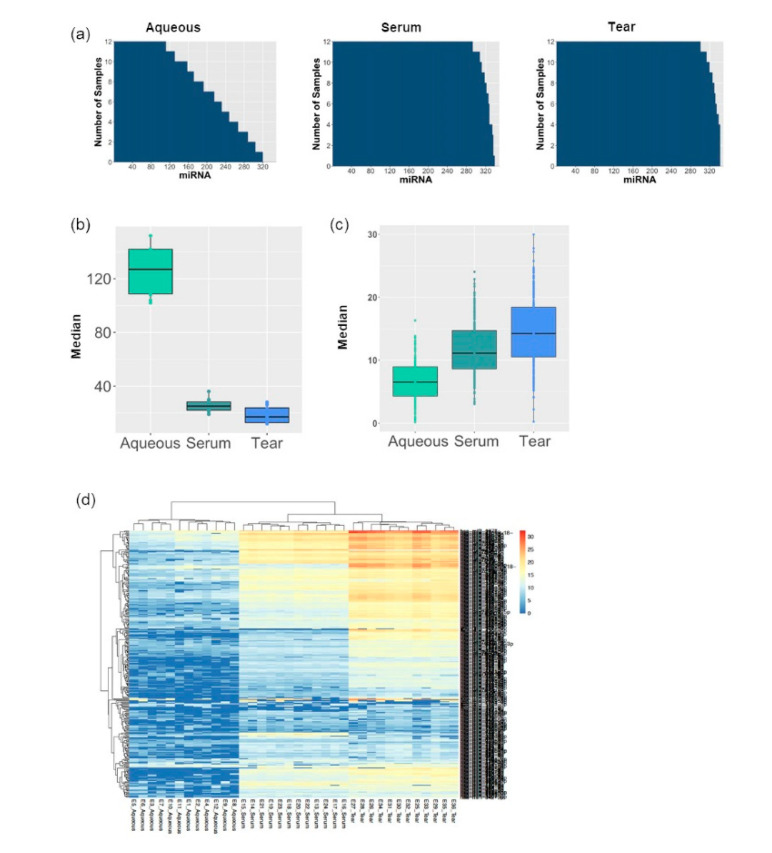
MiRNAs are more abundant in tear fluid in comparison with aqueous: (**a**) Barplots showing the number of miRNAs detected in each sample. More miRNAs were detected in tear and serum samples compared with aqueous. Aqueous samples showed large variation on the number of miRNA from 100 to 320; (**b**) Boxplot showing that tear samples had fewer nondetects compared with the other two biofluids; (**c**) Boxplot showing the median copy of miRNAs in each biofluid; (**d**) Heatmap comparing global mean normalized miRNA copies across all the three biofluids. Tear samples had the highest copy of miRNAs, while majority of the miRNA copy in aqueous was below 10.

The number of miRNAs detected in each sample was analyzed. Of the three biofluids, the largest number of miRNAs can be detected from tears (331), followed by serum (327) and aqueous (231) (Figure 1a). In serum and tears samples, a minimum number of 292 miRNAs and 300 miRNAs can be detected in all 12 samples, respectively. This is in contrast to 111 miRNAs detected in all 12 aqueous samples. Consistent with this, the mean copy number of miRNA within each biofluid was 11.36 in tears, 11.12 in serum, and 7.57 in aqueous (Figure 1c), and the absolute number of nondetects was the highest in aqueous (152) compared to serum (36) and tears (28) (Figure 1b). Next, the global mean normalized miRNA copy profiles for all three types of fluid samples were plotted on a heatmap (Figure 1d). Consistently, tears had the highest copy for majority of the miRNAs compared to serum and aqueous. These results indicate tear fluids contain the highest abundance of miRNA, which makes it a suitable biofluid for miRNA profiling and biomarker discovery. This is in contrast to aqueous humor, which has low miRNA copies with large intersample variation, rendering it an unsuitable candidate for biomarker discovery.

### 3.2. Tear Fluids miRNA Profiling Were More Consistent and Reproducible Compared to Aqueous Humor

**Figure 2:**
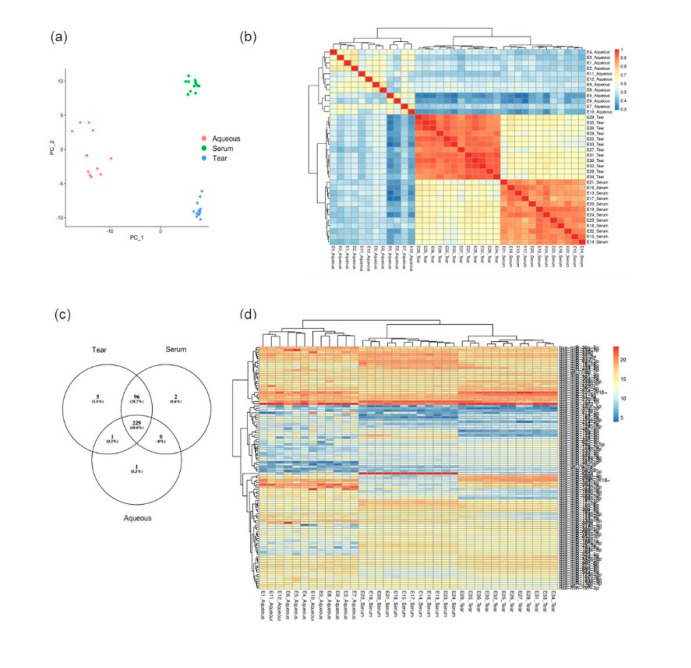
Good reproducibility and larger spectrum of miRNA profiling in tears: (**a**) Principal Component Analysis (PCA) on the miRNA showing that the biofluid type was the major source of the variation. The aqueous cluster showed much larger intragroup variation compared with tears and serum; (**b**) Heatmap showing Pearson correlation among the miRNA profiles from each sample. Tears and serum showed good correlations that were more than 90%, while aqueous showed poor correlations that were between 40–70%; (**c**) Venn diagram showing the overlaps in miRNA detected in the three biofluids. Tear miRNA spectrum greatly overlapped with serum and aqueous; (**d**) Heatmap showing the relative expression level of miRNAs detected in all the three types of biofluid. The miRNA copy was further normalized with quantile normalization cross the three types of biofluid and plotted as heatmap. The miRNAs in the top ranking showed similar profile between tears and aqueous.

To further evaluate if tear fluid is an ideal candidate for miRNA profiling for eye diseases, interpatient variation within each subgroup of biofluid and correlation across all three types of biofluids were analyzed. As shown in the PCA plot (Figure 2a), tight clusters formed in both serum and tear samples, but not in aqueous samples. This was due to the large intersample miRNA variation in aqueous samples secondary to (1) the low absolute copy number of miRNAs and (2) a high number of nondetects. Limitations in the dynamic range of miRNA quantification may have also been a contributing factor. In addition, Pearson correlation analysis (Figure 2b) showed a strong correlation in tear fluids (>90%) but a weak correlation in aqueous humor (40% to 70%). This further supports the low reproducibility of the miRNA data set derived from aqueous samples observed in Figure 2a. On the contrary, tears have the lowest inter- and intrapatient variation and highest reproducibility, rendering it an ideal biofluid for biomarker discovery.

To analyze the similarity of the miRNA profiles across tears, aqueous, and serum, Venn diagram analysis was performed (Figure 2c). Out of a total of 327 miRNAs in serum and 331 miRNAs in tears, respectively, 325 miRNAs were commonly detected in both serum and tears. Importantly, 99% of miRNAs detected in aqueous humor were also identified in tears (i.e., 230 out of 231), and only 1 miRNA was unique to aqueous alone (Figure 2c). This suggests that a majority of the miRNA detected in aqueous was a subset of that identified in tears. Interestingly, the miRNA identified uniquely in aqueous was hsa-miR-431-3p, which regulates the expression of the mTORC1 protein complex involved in inflammation. The miRNAs uniquely identified in serum were hsa-miR-208a-3p and hsa-miR-144-3p. The miRNAs uniquely detected in tears were hsa-miR-330-5p, hsa-miR-218-5p, hsa-miR-195-3p, hsa-miR-1471, and hsa-miR-767-5p. Of note, hsa-miR-330-5p and hsa-miR-218-5p (uniquely identified in tears) are both involved in the VEGF receptor-2 (VEGFR2) signaling pathway.

Quantile normalization was performed to minimize batch to batch variations arising from technical processing of RT-qPCR data and to allow the miRNA quantification comparable across biofluid types with different miRNA abundancy. The normalized miRNA expression was plotted on a heatmap (Figure 2d). The top-ranking miRNAs showed a similar pattern across the three biofluids, indicating tears may partially represent the miRNA profile in aqueous to a certain degree.

### 3.3. Distinct miRNA Expression Signatures in Tear Were Associated with Patient Response to Anti-VEGF Treatment

**Figure 3:**
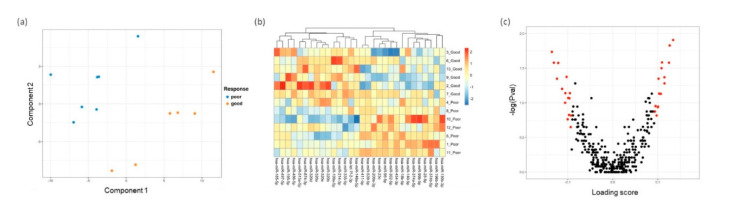
Differential miRNA expression analysis in tears samples according to the patients’ response to anti-VEGF treatment. Tears samples were categorized into Poor (poor responders), Partial (partial responders), and Good (good responders) according to the patients’ response to anti-VEGF treatment: (**a**) sPLS-DA analysis on the similarity of the samples was done based on the grouping Poor vs Good; (**b**) Heatmap showing the differentially expressed miRNAs with the sPLS-DA loading scores. The miRNAs form clear clusters according to high expression in Good or Poor; (**c**) Volcano plot showing the *p*-values calculated using Wilcoxon test and plotted against loading scores from sPLS-DA for all miRNAs. The differentially expressed miRNAs shortlisted from sPLS-DA analysis are highlighted in Red. Highly positive loading scores indicate higher expression in Good, whereas highly negative loading scores indicate higher expression in Poor.

Tears samples for all 24 patients collected at baseline (prior to treatment) were analyzed to identify distinct miRNA expression that can predict response to three consecutive anti-VEGF treatments. The patient demographics and clinical and ocular characteristics are shown in Table 1. Patients with DME were grouped into good responders, partial responders, and poor responders based on their assessment on OCT [32]. When tears samples were collected at baseline (i.e., prior to initiation of treatment) from all six good responders, eight partial responders, and ten poor responders were included in the initial differential miRNA analysis using sPLS-DA analysis (Figure S1A), they did not segregate into clear clusters. Similarly, heatmap analysis did not reveal a sub-group of differentially expressed miRNA (Figure S1B). In contrast, a further subgroup analysis inclusive of only six good responders and ten poor responders (i.e., two extremes of response stratification) revealed that good responders segregated well from the poor responders (Figure 3a). The heatmap of the sPLS-DA loading scores shows clear segregation of the 30 miRNAs that were differentially expressed between good and poor responders (Figure 3b), with 15 miRNAs highly expressed in either good or poor responders. The volcano plot in Figure 3c shows the *p*-values from the Wilcoxon test against the loading scores from sPLS-DA for each miRNA. Of these 30 miRNAs, 12 showed significant *p*-values (<0.05), and the remaining 18 had *p*-values lower than 0.2.

**Figure 4:**
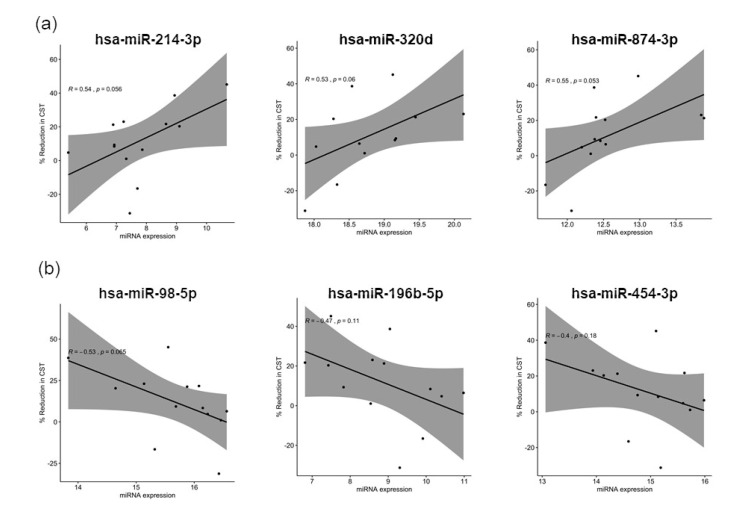
The correlation between the candidate miRNAs expression and the patients CST changed after the anti-VEGF treatment. Scatterplots showing the correlation between the miRNA loading score from sPLS-DA and the percentage reduction in CST changes. *R* values show Pearson correlation coefficient and *p*-values evaluates trend line fit: (**a**) The top three miRNAs in good responders showed positive correlation with the percentage reduction of CST; (**b**) The top three miRNAs in poor responders showed negative correlation with the percentage reduction of CST.

To verify if the 30 shortlisted miRNAs are able to predict the response to anti-VEGF treatment, Pearson correlation values were calculated for miRNA loading scores from sPLS-DA against the percentage reduction in central subfield thickness (Table 2). Figure 4a shows the top three miRNAs that had positive Pearson correlation value in good responders: hsa-miR-214-3p (*R* = 0.54, *p* = 0.056), hsa-miR-320d (*R* = 0.53, *p* = 0.06), and hsa-miR-874-3p (*R* = 0.55, *p* = 0.053). Similarly, Figure 4b shows top three miRNAs had negative Pearson correlation value in poor responders: hsa-miR-98-5p (*R* = −0.53, *p* = 0.065), hsa-miR-196b-5p (*R* = −0.47, *p* = 0.11), and has-miR-454-3p (*R* = −0.4, *p* = 0.18).

### 3.4. Differentially Expressed miRNA Targeting Genes and Functional Analysis

**Figure 5:**
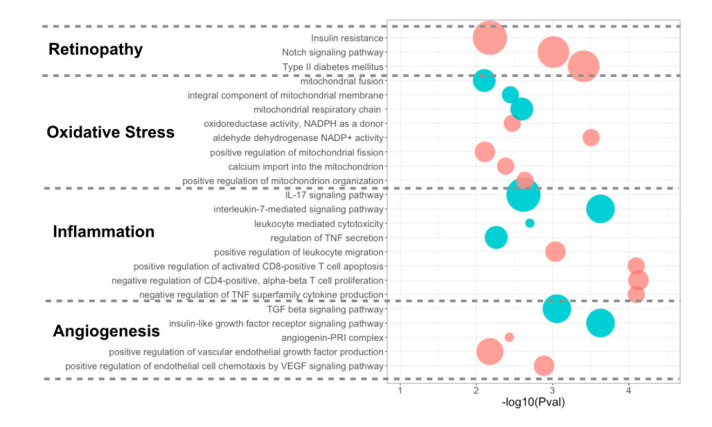
Signaling pathway enrichment for the 30 miRNAs identified between Good and Poor. The enriched pathways related to DME pathogenesis are categorized into angiogenesis, inflammation, oxidative stress, and retinopathy. These were plotted onto a scatterplot based on the *p*-values and the number of miRNAs in each pathway. Pathways enriched in the miRNAs highly expressed in Good are colored red, and those enriched in miRNAs highly expressed in the Poor are colored blue. The area of the circle is proportional to the number of miRNAs predicted to target the pathway.

MiRNAs primarily repress gene expression through regulation at both the level of mRNA stability by conducting mRNA degradation and the level of translation at initiation and after initiation. MiRNAs inhibit protein translation by degrading the polypeptides through binding complementarily to 3′UTR of the target mRNAs. To explore the various miRNA targets and their associated biological function and pathways, Gene Ontology (GO) and KEGG miRNA pathway enrichment analysis were applied to these 30 miRNAs. In particular, the angiogenesis, inflammation, and oxidative stress pathways were focused on due to their relevance to DME pathogenesis (Figure 5). A scatterplot was generated for the GO terms and pathways enrichment in both good responders and poor responders (Figure 5). Interestingly, VEGF-related pathways and the angiogenin-PRI complex that triggers angiogenesis were enriched in the good responders, while the transforming growth factor-β (TGF-β) and insulin-like growth factor (IGF) pathways that stimulate vasculogenesis were enriched in the poor responders. Additionally, both good and poor responder groups were enriched with inflammation-related pathways (TNF-α pathway, and pathways regulating leukocyte recruitments and cytokines secretion) and mitochondria-related oxidative stress (Figure 5).

**Figure 6:**
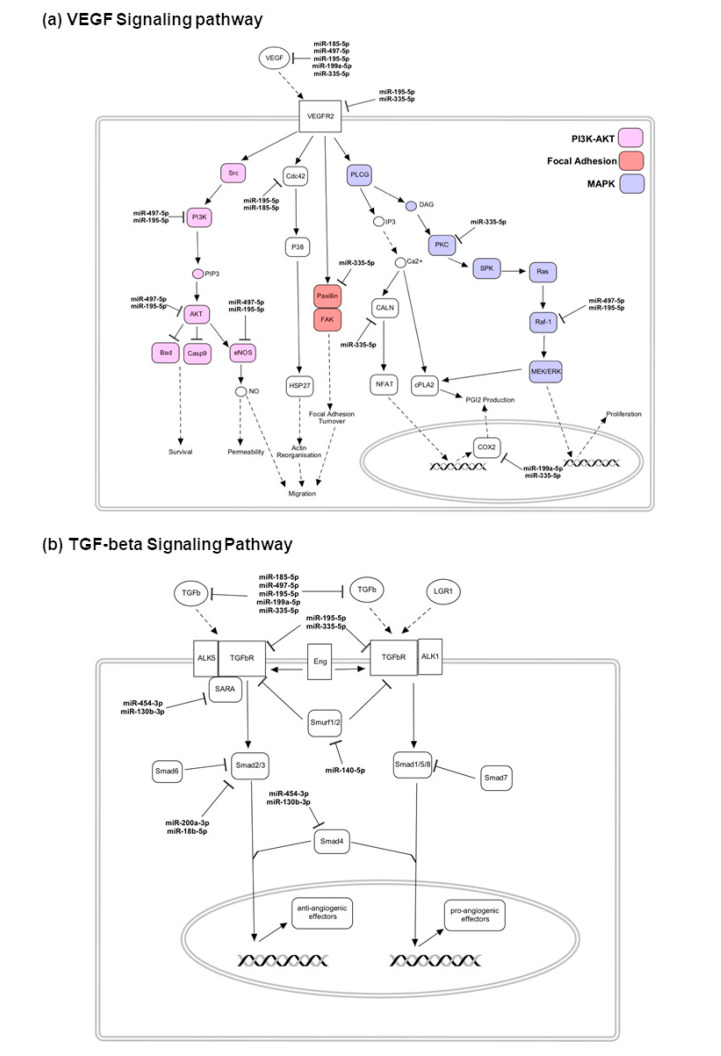
KEGG Signaling Pathways involved in angiogenesis targeted by differentially expressed miRNAs: (**a**) VEGF signaling pathway components targeted by miRNAs upregulated in good responders; (**b**) TGF-beta signaling pathway components targeted by miRNAs upregulated in poor responders.

To better understand the role of these 30 miRNAs in the main angiogenesis pathways, such as the VEGF, IGF, and TGF-β pathway, the miRNAs were mapped to their target genes within their respective pathways (Figure 6 and Figure S2). In good responders, seven of eight miRNAs enriched in the VEGF signaling pathways inhibited the VEGF and VEGFR expression (Figure 6a). In addition, key genes involved in the downstream pathways of VEGF, such as PI3K, MAPK, and focal adhesion signaling pathways, were targeted by miRNAs in good responders (Figure 6a). This may indicate that the VEGF signaling pathway was already downregulated by the presence of eight miRNAs prior to the initiation of treatment, rendering them more sensitive to anti-VEGF treatment. In contrast, both TGF-β and IGF signaling pathways were enriched in poor responders (Figure 6b and Figure S2). Specifically, in the TGF-β pathway, both TGF-β and TGF-β receptors complex were repressed by miRNAs in poor responders. Key players such as SARA and Smad2/3/4 in the anti-angiogenic signaling cascades were targeted by miRNAs, while key players such as LRG1 and Smad1/5/8 were involved in pro-angiogenesis signaling cascades that were not targeted (Figure 6b). The relative suppression of the anti-angiogenic cascade (via Smad2/3) compared to the pro-angiogenic cascade (via Smad1/5/8) might account for the poor response of these patients to anti-VEGF treatment. Interestingly, in poor responders, all nine miRNAs targeted IGF-1 and IGF-1R from the IGF signaling pathways (Figure S2). This is in contrast to good responders, whereby miRNAs principally targeted the VEGF signaling pathways. This might account for why poor responders were less sensitive to anti-VEGF agents.

## 4. Discussion

This exploratory study compared the differential expression of miRNA biomarkers across three different biological fluids (tears, aqueous, and serum) from patients with DME. Tear fluid had the highest number of detectable miRNA species with good data reproducibility. After identifying 30 miRNAs in tear fluid with differential expression between good and poor responders to anti-VEGF therapy, in silico analysis predicted which pathways were upregulated in poor responders compared to good responders and offered mechanistic insight.

### 4.1. Extracellular miRNAs as a Biomarker in DME

A diagnostic biomarker able to determine anti-VEGF response in DR, specifically DME, with high specificity and sensitivity, would address an unmet clinical need. Important features of a good biomarker include stability, sensitivity, specificity, and easy obtainability, preferably via a noninvasive approach. Extracellular miRNAs are remarkably stable (under both standard and extreme conditions), ubiquitously expressed in biological fluids, involved in specific biological pathways, and easily detectable using relatively inexpensive assays. Studies investigating the role of circulating miRNAs in the pathogenesis of DR have identified a series of miRNAs that are up- or downregulated in murine retina and retinal endothelial cells [29,42,43]. However, to date, dysregulation of miRNA expression has been determined for DR but not specifically for DME. This study successfully identified differentially expressed miRNAs in tear fluid between good and poor responders to anti-VEGF treatment in DME patients.

### 4.2. Comparison of the miRNA Expression Profile of Tear Fluid, Aqueous Humor, and Serum

Tear fluid had the largest number and higher concentration of detectable miRNAs, followed by serum, then aqueous. Our findings of a comprehensive miRNA repertoire in tear fluid are consistent with a previous report which isolated 320 miRNAs from tears [15]. In our study, aqueous humor had 115 fewer miRNA species than tear fluid and more interindividual variation than the other two biofluids. The identified issues of lower miRNA abundance and higher sample variability in aqueous humor were also found in another study that used qPCR arrays [44].

### 4.3. Upregulated miRNAs and the Targeting Pathways in Good Responders

In good responders, the VEGF and Notch signaling pathways were targeted by the miRNAs (Figure 5), and these pathways have been described to interact dynamically at the cellular level to control the vascular network [45].

The pathogenesis of DME is intimately related to VEGF-mediated dysregulation of vascular permeability. Detailed analysis of the published literature has shown that in vivo and in vitro VEGF-mediated permeability differ in time course, but have common involvement of many specific signaling pathways, particularly VEGF receptor-2 (VEGF-R2) activation, calcium influx through transient receptor potential channels, activation of phospholipase C gamma, and downstream activation of nitric oxide synthase. Pathways downstream of endothelial nitric oxide synthase appears to involve the guanylyl cyclase-mediated activation of the Rho–Rac pathway, and subsequent involvement of junctional signaling proteins, such as vascular endothelial cadherin and the tight junctional proteins zona occludens (ZO) and occludin, are linked to the actin cytoskeleton [46]. Endothelial cells of the retinal capillary plexuses are firmly connected by tight and adherent junctions, forming the endothelial cell-tight junction (EC-TJ) complexes which constitute the inner blood–retina barrier (iBRB). Upregulation of VEGF-A induces phosphorylation of occludin and ZO1 and disassembly of the TJ proteins leading to the dysregulation of EC paracellular permeability [47,48,49]. Additionally, high levels of VEGF increase the expression of intercellular adhesion molecule I (ICAM-1), causing leucocyte adherence to the retinal endothelium, endothelial cell death, and, ultimately, vascular leakage.

The Notch pathway regulates developmental angiogenesis as well as post-angiogenic blood vessel remodeling and homeostasis [45,50]. Elevated Notch1 ligands are present in the vitreous of a subset of patients with DME [50]. Perturbed Notch1 signaling has been found to disrupt endothelial junctions by causing dissociation of vascular endothelial-cadherin from β-catenin, and the neutralization of Notch1 ligands decreases pathological vascular permeability in diabetic retinas [50]. However, Notch signaling is also important for pericyte stability and survival [51,52]. Additionally, miRNAs targeting the angiogenin-PRI (human placental ribonuclease inhibitor) complex were also upregulated in good responders. Angiogenin (ANG) is a multifunctional proangiogenic protein that induces blood vessel formation in both physiological and pathological processes, while PRI abolishes its angiogenic activity [53]. ANG binds to membrane surface actin of vascular endothelial cells, triggering endothelial cell proliferation and mature new blood vessel formation. Studies evaluating the serum concentration of ANG in T1DM and T2DM patients have shown contrasting results, suggesting a difference in the angiogenic process between both types of DM [54,55,56]. Specific to diabetic retinopathy (DR), a study comparing serum and vitreous concentration of angiogenin versus VEGF showed a two-fold reduction in vitreous and serum angiogenin concentration in DR patients compared to those without DR while the reverse is seen with VEGF [57]. These findings suggest that angiogenesis in DR is primarily VEGF-driven. However, the precise role of angiogenin-PRI complex in DR and DME remains to be elucidated.

We report, for the first time, an increased expression of miR-214-3p, miR-320d, and miR-874-3p in patients with DME, and a positive Pearson correlation in relation to CST reduction in response to anti-VEGF treatment. Increased expression of miR-214 has been identified in diabetic patients [58,59] and in a mouse model of retinal ischemia [60]. Studies of miRNA-214 in vitro have demonstrated its role in the suppression oxidative stress in diabetic nephropathy [61] and in the repression of the senescence of endothelial cells [62]. Increased expression of miR-320a and miR-320b has been identified in eyes with proliferative diabetic retinopathy [63], but this is the first report of an association between miR-320d and diabetic retinopathy. MiR-874 has been found to alleviate diabetic retinopathy in a murine model by regulating the NF-κB signaling pathway [64], ameliorating renal injury in diabetic nephropathy [65], and providing neuroprotection in a stroke model [66].

### 4.4. Upregulated miRNAs and the Targeting Pathways in Poor Responders

Of particular interest are pathways that are targeted by miRNAs highly expressed in poor responders to anti-VEGF treatment, as this may elucidate other mechanisms of DME that are not mediated by VEGF and identify new treatment targets. The identified pathways included insulin-like growth factor (IGF) receptor and transforming growth factor-beta (TGF-β).

In DR, overexpression of IGF-1 in the retina, along with increased IGF-1R mediated signaling, resulted in the accumulation of VEGF and upregulation of vascular intercellular adhesion molecule I (ICAM-1). Together, these changes disrupt the vascular tight junction integrity, causing the breakdown of the inner blood–retinal barrier and DME. In this study, selective targeting of the IGF-1 signaling pathway (Figure S2) specific to the poor responders suggests that alternate (non-VEGF) treatment targets should be considered.

The TGF-β signaling pathway (Figure 6b) was also preferentially targeted in poor responders. TGF-β signaling is important in both physiological and pathological angiogenesis. TGF-β signals via TGF-βRI (ALK-5) and TGF-βRII (ALK-1) receptors, both of which are expressed in the retinal endothelial cells. Retinal endothelial cells can respond to TGF-β via two pathways: (1) Stimulation of angiogenesis through upregulation of ALK1-Smad1/5/8, or (2) inhibition of angiogenesis via ALK5-Smad2/3/4 signaling. Interestingly, our findings among poor responders showed selective targeting of the anti-angiogenic pathway (ALK5-Smad2/3/4) and sparing of the pro-angiogenic pathway (ALK1-Smad1/5/8). Therefore, it is tempting to speculate that the pro-angiogenic arm of the TGF-β signaling pathway can be another potential treatment target for poor responders.

Increased expression of miR-98-5p, miR-196b-5p, and miR-454-3p was found in DME patients with poor response to anti-VEGF treatment. MiR-98 is a stress-related microRNA that regulates oxidative stress and apoptosis [67,68], and negatively regulates tight junction-associated protein expression [69]. In mice with Alzheimer’s disease, miR-98 has also been found to inhibit the Notch pathway (discussed above) [70]. MiRNA-196b has been found to induce apoptosis in liver carcinoma cells by targeting insulin-like growth factor 2 RNA binding protein1 [71]. Increased expression of miR-454 has been identified in the serum of diabetes patients [72], and has been associated with the incidence and progression of diabetic retinopathy in type 1 diabetes [73]. Additionally, miR-454 has been found to suppress the secretion of VEGF in pancreatic cancer [74], and the expression of placental growth factor in human microvascular endothelial cells [75].

### 4.5. Limitations

The main limitation of this study was the small sample size, which may have led to an increased occurrence of false-positives as well as false-negative results. Another limitation was that a gender-stratified analysis was not performed, which may potentially have revealed a gender difference on the expression of certain miRNAs.

In Batch 2 of the study, patients with DME received either Bevacizumab or Aflibercept injection. Although both anti-angiogenic agents interact with VEGFA (VEGF165), thereby inhibiting angiogenesis, it is reasonable to argue that the intrinsic differences in the molecular structure, pharmacokinetics, and molar dose between both agents may therefore influence efficacy and response to treatment. Although Bevacizumab is deemed noninferior to Aflibercept, the two-year post-hoc area-under-the-curve analysis of DRCR.net Protocol T showed superior visual outcome with Aflibercept when compared to Bevacizumab and Ranibizumab particularly for DME eyes with worse initial visual acuity [76].

Compared to the vitreous, miRNAs found in tears may not be entirely representative of the milieu of retinal vascular diseases. Anatomically, the anterior hyaloid face of the vitreous is in communication with the aqueous humor. Therefore, we postulate that aqueous and vitreous humor may share a similar miRNA profile. In this study, nearly all miRNAs identified in aqueous were present in tears. Moreover, tear collection is a simple, noninvasive procedure in contrast with aqueous or vitreous sampling. Hence, it is reasonable to use tears as a surrogate.

## 5. Conclusions

This study attempted to address the unmet clinical need for a diagnostic biomarker to predict response to anti-VEGF treatment in DME. We reported novel miRNAs and provided insight into biological pathways implicated in DME. Our study identified tears as a potential biofluid for the screening of circulating miRNA. Further validation studies in larger independent cohorts are needed to determine the predictive performance of these miRNA candidate biomarkers prior to establishing a panel for use in clinical practice.

## Figures and Tables

**Table 1:** Patient demographics and clinical and ocular characteristics (*n* = 24).

Characteristics	Batch I	Batch II	
Treatment	Bevacizumab (*n* = 6)	Bevacizumab (*n* = 7)	Aflibercept (*n* = 11)
Age, years Mean ± SD	59 ± 8.7	57.6 ± 7.7	59 ± 10.0
Gender			
Male (%)	2 (33.3%)	6 (85.7%)	8 (72.7%)
Female (%)	4 (66.7%)	1 (14.3%)	3 (27.3%)
HbA1c Mean ± SD	8.0 ± 1.2	9.2 ± 2.0	7.5 ± 1.3
Central subfield thickness			
Baseline mean ± SD	349.7 ± 41.3	404.4 ± 94.6	458.3 ± 87.4
Post-treatment mean ± SD	324.2 ± 65.0	376.3 ± 106.3	369.3 ± 56.3
Response			
Good (%)	0 (0.0%)	2 (28.6%)	4 (36.4%)
Partial (%)	3 (50.0%)	2 (28.6%)	3 (27.2%)
Poor (%)	3 (50.0%)	3 (42.9%)	4 (36.4%)

**Table 2:** Summary of Pearson correlation between the 30 miRNAs loading scores from sPLS-DA and the percentage reduction in CST after anti-VEGF treatment. MiRNAs highly expressed in Good are shown in Red table, miRNAs highly expressed in Poor are shown in Blue table.

miRNA	*p*-Value	*R*-Value	miRNA	*p*-Value	*R*-Value
hsa-miR-148A-5p	0.48	0.2	hsa-miR-130b-3p	0.49	−0.12
hsa- let-7f-2-3p	0.15	0.34	hsa-miR-140-5p	0.78	0.83
hsa-miR-185-5p	0.2	0.4	hsa-mIR-18b-5p	0.097	−0.39
hsa-miR-195-5p	0.9	0.1	hsa-miR-196b-5p	0.12	−0.47
hsa-mIR-199a-5p	0.29	0.34	hsa-miR-200a-3p	0.61	−0.2
ho-miR-214-3p	0.13	0.54	hsa-miR-23c	0.25	−0.33
hsa-miR-320a	0.11	0.42	hsa-miR-28-5p	0.23	−0.35
hsa-miR-320b	0.06	0.51	hsa-miR-362-5p	0.19	−0.34
hsa-miR-320d	0.038	0.53	hsa-mIR-374a-5p	0.46	−0.25
hsa-miR-320e	0.055	0.52	hsa-miR-374b-5p	0.75	−0.13
hsa-mIR-335-5p	0.19	0.38	hsa-miR-411-5p	0.18	−0.38
hsa-miR-486-5p	0.15	0.48	hsa-miR-454-3p	0.15	−0.4
hsa-miR-497-5p	0.23	0.35	hsa-mIR-539-5p	0.25	−0.34
hsa-miR-513a-5p	0.11	0.46	hsa-miR-98-5p	0.049	−0.53
hsa-mIR-874-3p	0.048	0.55	hsa-miR-99b-5p	0.27	−0.34

## Supplementary Materials

The following are available online at https://www.mdpi.com/2077-0383/9/9/2920/s1, Figure S1: Differential miRNA expression analysis in tears samples according to the patients’ response to anti-VEGF treatment, Figure S2: IGF pathway signaling components targeted by miRNAs upregulated in poor responders.
