# Supplementary figures and images for: A Pilot Study on MicroRNA Profile in Tear Fluid to Predict Response to Anti-VEGF Treatments for Diabetic Macular Edema
Source: J Clin Med. 2020 Sep 10;9(9):2920. doi: 10.3390/jcm9092920 (PMC7564365; doi:10.3390/jcm9092920)

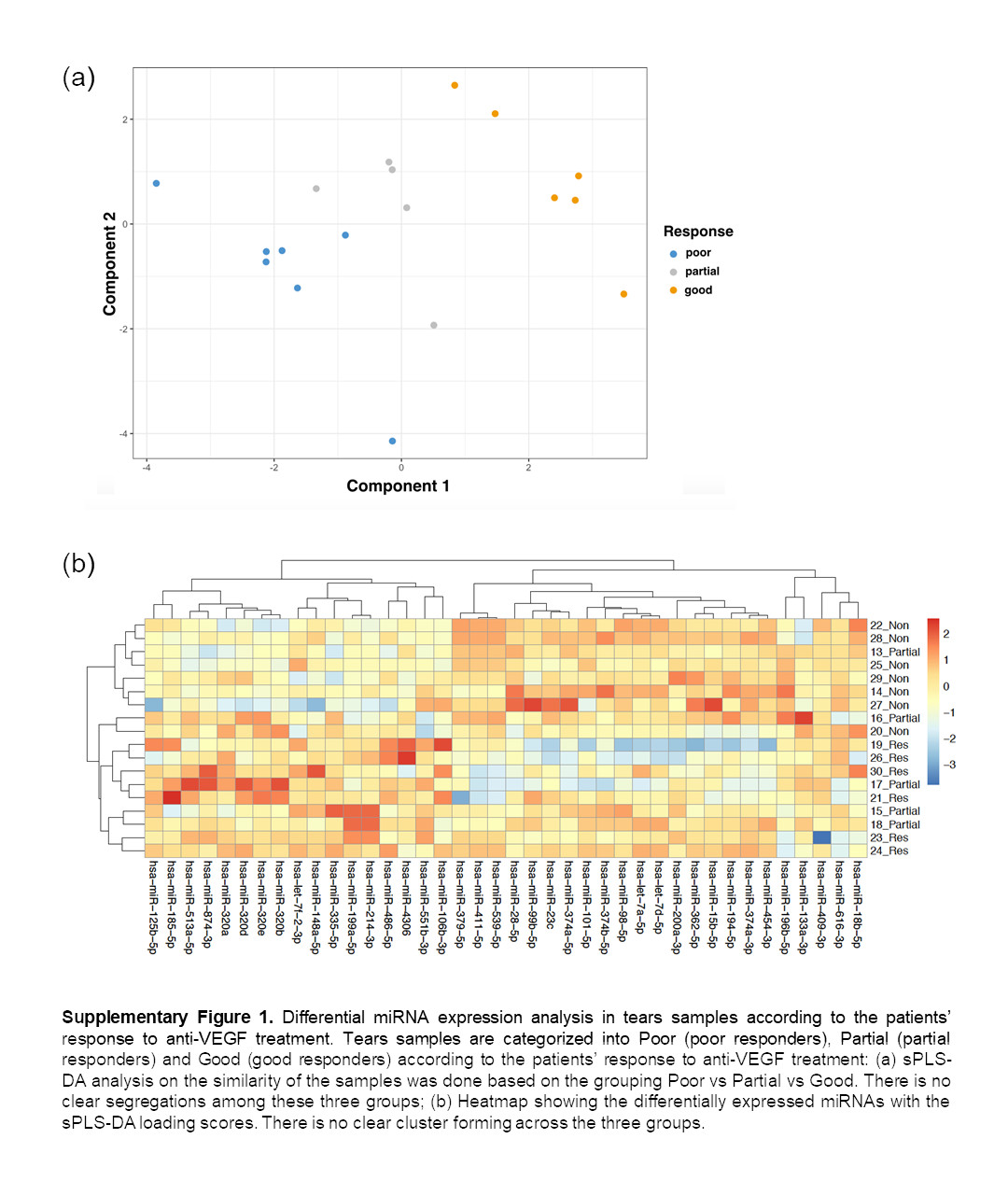

Supplement: Supplementary file 1 [file jcm-09-02920-s001.zip › Supplementary Figure 1.jpg]

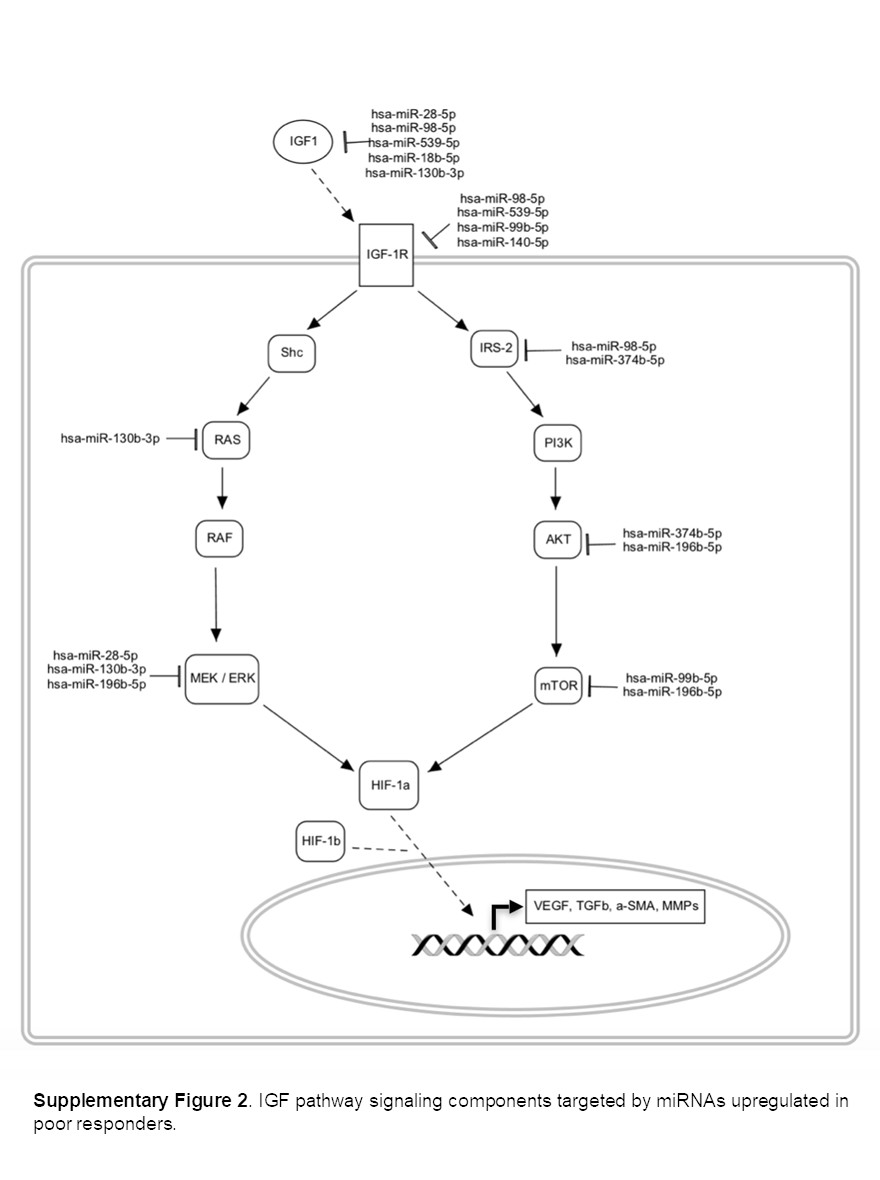

Supplement: Supplementary file 1 [file jcm-09-02920-s001.zip › Supplementary Figure 2.jpg]
